# Statistical analysis plan and protocol updates for Gestational diabetes in Uganda and India: Design and Evaluation of Educational Films for Improving Screening and Self-management (GUIDES) trial

**DOI:** 10.1186/s13063-023-07508-5

**Published:** 2023-08-12

**Authors:** Nick Birk, Laura L. Oakley, Poppy A. C. Mallinson, Deepa R, Giridhara R. Babu, Moffat Nyirenda, Sanjay Kinra

**Affiliations:** 1 Department of Non-Communicable Disease Epidemiology, London School of Hygiene and Tropical Medicine, Keppel Street, London, WC1E 7HT UK; 2 Centre for Fertility and Health, Norwegian Institute of Public Health, Skøyen, P.O. box 222, N-0213 Oslo, Norway; 3 Indian Institute of Public Health-Bengaluru, Public Health Foundation of India (PHFI), Bengaluru, India; 4 MRC/UVRI and LSHTM Uganda Research Unit, Entebbe, Uganda

**Keywords:** Statistical analysis plan, Gestational diabetes, Behavioural intervention, Cluster randomised trial

## Abstract

**Background:**

Timely detection and management of gestational diabetes mellitus (GDM) have been identified as a high priority for policymakers in low- and middle-income countries (LMICs). The GUIDES trial will evaluate a package of three interconnected film-based interventions aimed at improving the timely detection and management of GDM. The protocol for this trial has previously been published; this publication outlines the statistical analysis plan for the trial.

**Methods and design:**

The GUIDES study is a multi-country cluster-randomised controlled trial consisting of one trial conducted in Uganda and one in India (30 clusters in each country). Mixed effects models will be used to compare the primary study outcomes of the proportion of women who are tested for GDM between 24 and 32 weeks of pregnancy and the mean fasting blood sugar of women with GDM at 34-week follow-up while accounting for clustering. Secondary analyses will compare the proportion of women with self-reported GDM diagnosis at 32 weeks of pregnancy and the proportion of women with adverse perinatal outcomes related to GDM up to 4 weeks after birth in each trial arm.

**Trial status and discussion:**

Follow-up is expected to end in March 2023 in Uganda and in May 2023 in India. Analyses will be carried out following this statistical analysis plan in the month following trial completion.

**Trial registration:**

ClinicalTrials.gov NCT03937050. Registered on 3 May 2019. Clinical Trials Registry India CTRI/2020/02/023605. Registered on 26 February 2020.

## Background

Women diagnosed with gestational diabetes mellitus (GDM) have a ~ 50% greater risk of developing type 2 diabetes mellitus in later life, and the risk of obesity and diabetes among their offspring may also be increased [1, 2]. In the short term, GDM is associated with a higher risk of adverse perinatal outcomes such as stillbirth and birth complications [3]. Timely detection and management of GDM have been identified as a high priority for policymakers in LMICs [4–6].

The original study protocol has been published elsewhere [7]. In summary, this trial will evaluate a package of three interconnected educational/behavioural interventions aimed at (a) improving skills and knowledge of GDM guidelines among health providers (b) raising awareness of the importance of GDM screening among pregnant women and their families and (c) improving confidence and skills in self-management among those diagnosed with GDM. The interventions will be delivered through the medium of film and evaluated through two independent trials in Wakiso, Mpigi and Masaka, Uganda, and Bengaluru, India.

Our aim is to assess whether an educational/behavioural intervention delivered through a package of culturally tailored films for pregnant women, their family members and health providers can improve timely detection, glycaemic control and clinical outcomes of women with GDM. The specific objective of this trial is to evaluate the effectiveness of the intervention (i.e. combined package of GDM films) in improving timely screening and detection of GDM, decreasing the incidence of adverse GDM-related perinatal outcomes and, among women with GDM, to improve glycaemic control.

## Methods and design

### Trial design

The GUIDES study is a multi-country cluster-randomised controlled trial with a parallel group design. It consists of two trials, one being conducted in Uganda and one in India. Randomisation occurred at the level of the health centre with a 1:1 allocation of health centres to intervention:control within each country. The trial is open-label, and the data will be analysed at the individual (rather than cluster) level.

### Participating facilities

Sixty clinic facilities meeting the inclusion criteria (government-funded health centres recording a minimum of 200 births per year) were recruited for the trial: 30 in Uganda (Wakiso, Masaka and Mpigi districts) and 30 in India (urban Bengaluru).

### Randomisation

The 60 clinics were defined as clusters. Randomisation was carried out separately for each country, with clusters assigned to intervention or control in a 1:1 ratio. Covariate-constrained randomisation was used to help ensure balance with respect to the following covariates: size of facility (as determined by number of deliveries per year); health facility (HF) level (levels I, II and III in India or level III or IV in Uganda) and urban/peri-urban or rural setting (Uganda only). Randomisation was implemented by an independent trial statistician using the *cvcrand* command in Stata. Concealment of allocation was ensured because the sites were enrolled before the randomisation was conducted. Group allocation was not blinded to researchers, site staff or participants. Data analysts will be blinded to the allocation group of individual sites until the analysis code is finalised.

### Recruitment of participants

Women were eligible for the study if they were 18 years of age or older, were of gestational age less than 32 weeks at recruitment, were able to provide informed consent and were available for follow-up for the study duration. Prospective participants receiving antenatal care at participating clinics were made aware of the study through posters and information sheets. Country-specific recruitment protocols were as follows.

#### UGANDA

Pregnant women attending participating health clinics for their first antenatal visit were invited to take part. Local fieldworkers visited clinics, distributed the patient information sheet (PIS) and obtained written informed consent for study participation. Once consent was provided, women were asked to complete a short questionnaire and to give contact details.

#### INDIA

Pregnant women potentially eligible for recruitment were identified via the Bruhat Bangalore Municipal Corporation (BBMP), which provided regular updates on women registering for antenatal care at participating clinics. Remote recruitment was used: fieldworkers contacted potentially eligible women, explained the study, confirmed eligibility and assessed interest in participation. The PIS was sent electronically (via WhatsApp or similar), and electronic consent (eConsent) was sought using a Google Docs form. For women subsequently diagnosed with GDM who attended a clinic visit at 34 weeks, written informed consent was added at this time.

Between April and December 2022, the study launched a targeted recruitment drive in India to focus on pregnant women with GDM. Research assistants identified women with GDM (defined as 140 mg/dL glucose value following oral glucose challenge test (OGCT)) by contacting local laboratories and tertiary and referral hospitals in the study area. Fieldworkers contacted by phone women who could be linked to a participating study facility (registered and obtained from their maternal and child health services card). The existing study procedures (sharing of PIS, obtaining consent, data collection) were followed for women with GDM identified via this strategy. Women with GDM who were receiving care at (or were assigned to) an intervention facility were invited to receive the self-management intervention either in-person or remotely, as described in the protocol.

### Withdrawal

Participants are welcome to withdraw from the study at any time without giving reasons. The local investigators and PI also have the right to withdraw patients from the study in the event of illness, protocol violations or for administrative or other reasons. The information already observed will be kept on these participants, but they will not be contacted for further follow-up and data collection. Loss to follow-up at each time point will be included in the CONSORT flow diagram [8]. The number of individuals who withdraw due to adverse events will be reported. The baseline demographic characteristics of women who were lost to follow-up will be summarised in a table and compared to the baseline demographic characteristics of women who completed the trial.

### Study objectives

The primary study outcomes are as follows:

- The proportion of women who are tested for GDM between 24 and 32 weeks (6 and 8 months) of pregnancy (self-reported at 32 weeks) (co-primary outcome 1)
- The mean fasting blood sugar (mmol/L) of women with GDM at 34-week follow-up (co-primary outcome 2)

The secondary study outcomes are as follows:

- The proportion of women with self-reported GDM diagnosis at 32 weeks (secondary outcome 1)
- Proportion of women with adverse perinatal outcomes related to GDM (self-reported composite of emergency caesarean section delivery, perinatal or neonatal mortality and infant hospitalisation within 4 weeks of delivery) (secondary outcome 2)

### Timing of analyses and outcomes

No interim analyses or stopping guidance were planned for this trial as the intervention is low-risk. However, the aggregate prevalence of GDM screening and self-reported diagnosis were regularly checked to understand overall trends in each country.

Final analyses are set to begin within 1 month of all follow-up data collection being completed across both sites. Follow-up is anticipated to conclude in May 2023 in India and in March 2023 in Uganda.

GDM screening will be assessed at a 32-week follow-up (gestational age). If a woman is nearly 32 weeks into her pregnancy at baseline, a minimum of 1 week must pass between the baseline assessment and the first follow-up. The fasting blood sugar of women with GDM will be assessed at a 34-week follow-up (gestational age). The presence of adverse perinatal outcomes related to GDM will be reported at the final follow-up scheduled to occur 6 weeks after birth or at the 32-week follow-up if the woman reports she is no longer pregnant at this time.

### Sample size

Based on pilot data from Bengaluru, we anticipated that we would be able to recruit approximately 10,000 pregnant women in each country during a 1-year recruitment period (an average of one delivery per day per unit). Of these, we expected that approximately 10% (*n* = 500) in the intervention arm, and 5% (*n* = 250) in the control arm would be diagnosed with GDM. However, data monitoring revealed that the overall prevalence of self-reported GDM was less than 5% in both countries, suggesting a lower sample size of women with GDM given the same overall recruitment rate.

Our initial estimated sample size requirements (per site) at the outset of the trial were 1218 pregnant women for the original co-primary outcome of GDM detection (5% vs 10%) [9] and 5935 women for the secondary outcome of composite adverse perinatal outcomes (30% vs 35%) [10, 11]. However, as the prevalence of the new co-primary outcome is notably different than the original co-primary outcome, the sample size calculations were re-computed for the new co-primary outcome of timely GDM screening. Under the conservative assumption that the intervention would increase timely GDM screening by a minimum of 10% (based upon three previous trials of interventions to promote screening among women with GDM [12–14]), and considering the observed in-trial prevalence of timely screening in each setting (52.5% in Uganda and 33.5% in India), we anticipate that we will now need a minimum sample of 2220 women in Uganda and 1290 women in India for this revised outcome.

For co-primary outcome 2 of the mean fasting blood sugar at 34 weeks, the previous sample size calculation suggested a need to recruit at least 180 women with GDM (fasting glucose difference of 0.3 mmol/L, for an SD of 0.9 mmol/L) (5), which will still be met under the updated diagnosis rate assumptions (in which we expected at least 200 women with GDM in each site).

### Statistical analysis plan

The current document represents the most recent version of the statistical analysis plan (SAP) for GUIDES (version 2.1, 16 December 2022). The SAP was designed based on protocol v4.3, following the guidelines of Gamble et al. [15] and was finalised prior to the completion of trial follow-up data collection. Data have not yet been viewed disaggregated by the trial arm at the time of SAP completion.

### Descriptive analyses

Baseline patient characteristics will be presented by country in a tabular format. Though we will be collecting information about a variety of demographic and lifestyle factors, a subset of these variables will be presented in the main table, and full distributions will be described in supplementary material. Categorical variables will be presented as *n* (%), and continuous variables will be presented as mean (SD) or median (IQR) if the distribution is skewed (as determined by visual inspection). The variables to be reported in the main study table are participant age, gestational age at baseline in weeks, marital status, household size, income sufficiency, education, parity, previous obstetric history, current pregnancy risk factors, GDM-specific risk factors, physical activity and dietary behaviour. The number of women recruited from each health centre will also be reported.

To assess the representativeness of the trial sample, we will report the number of women who are assessed for eligibility, the number eligible and the number recruited in each clinic. Furthermore, we obtain otherwise eligible women’s reasons for not joining the trial, which may provide insight into any potential differences between the study population and the general population. A CONSORT flow diagram will be used to visualise the sample size at each of these stages and for each analysis endpoint.

### Analysis framework

The main analyses will be performed on an intention-to-treat basis using a superiority framework. That is, participants will be analysed according to the randomisation arm to which their health clinic was assigned, regardless of the level of engagement with the intervention materials. The analyses will be performed separately for each country. Analyses will be conducted using the R and Stata software.

### Primary analyses

The primary analysis for the prevalence of timely GDM screening will utilise a logistic mixed effects model for a binary outcome. The null hypothesis is that the proportion of women who self-report screening for GDM between 24 and 32 weeks is the same in both arms. The model will include a random effect for the health centre (unit of randomisation), a fixed effect for the treatment arm (exposure) and fixed effects for cluster-level characteristics used as strata in randomisation (i.e. clinic size, facility level and (in Uganda) urbanicity). We will report the model-based prevalence ratio and prevalence difference in the primary outcome between intervention and control arms, with appropriate 95% confidence intervals. As a sensitivity analysis, the results will also be reported using cluster-level summaries (adjusted for covariates as above).

For the co-primary outcome of glycaemic control, we recognise that the participants who are diagnosed with GDM in the two trial arms may not be comparable, as the intervention encourages more frequent screening. We will consider the use of propensity score adjustment to take account of this non-randomised comparison, if appropriate. Differences in baseline covariates related to glycaemic control will be assessed by comparing treatment arms for differences of clinically relevant magnitude. Indication of imbalance will prompt the use of these variables in the construction of the propensity scores. Covariates to be considered will include age, socioeconomic status, baby previously weighing more than 4 kg, previous GDM diagnosis, family history of diabetes and tobacco use. In the case that many variables are related to the exposure, we will utilise the statistical rule of thumb that suggests using no more than one covariate per every 10 individuals in the smaller arm. Variables will be assessed for inclusion in the propensity score model based on the level of statistical significance with which they are related to the trial arm. The mixed effects models described for the prevalence of GDM diagnosis will be used, though with a continuous, rather than binary, outcome specification. A plot will be generated to compare model residuals to fitted values to check that linear regression assumptions are satisfied and to identify outliers or points which are given disproportionate influence in fitting the model. Appropriate transformations may be made applied to the outcome variable as necessary, and if any substantial outliers are identified through visual inspection, the analysis will be repeated with outliers removed as a sensitivity analysis. If an indication of clinically significant imbalance had been noted, the propensity score will additionally be included in the model as a continuous covariate.

### Secondary analyses

The proportion of women with adverse perinatal outcomes related to GDM (self-reported composite of emergency caesarean section delivery, perinatal or neonatal mortality and infant hospitalisation within 4 weeks of delivery) will be compared between trial arms. As with the primary outcome of GDM diagnosis, this will be assessed through the use of logistic mixed effects models with a random effect for the health centre and fixed effects for the pre-specified cluster-level variables. The same statistical methods will also be used to assess the secondary outcome of self-reported GDM diagnosis. For these outcomes, we will also present models adjusted for key a priori individual-level confounders that are strongly associated with the outcomes, namely age, parity and education.

### Missing data

As the follow-up data are collected through telephone surveys, there is a risk that participants may not be contactable for follow-up. If less than 5% of women who participate at baseline are lost to follow-up or have missing data in an outcome variable for another reason, a complete case analysis will be used. If more than 5% of women who participate at baseline are lost to follow-up or have missing data on an outcome variable, we will perform multiple imputation based on available covariates (making the assumption that the data are missing at random (MAR)). We will use a multilevel imputation model to account for the lack of independence of observations from the same cluster. Appropriate confidence intervals will be computed [16]. To explore the sensitivity of these results, complete case analyses will also be presented. If more than 40% of women have missing data in variables needed to conduct a given analysis, pairwise deletion will be utilised [17].

### Adverse events

The proportion of women with adverse perinatal outcomes related to GDM is assessed as a secondary outcome of the trial. As the intervention is very low risk, we do not anticipate that any adverse events will be causally linked to the intervention. Further analyses of safety are likely to be underpowered since the sample size was not calibrated to detect an effect for these events, assuming that the rate of specific adverse events is less than that of the composite outcome of adverse perinatal events related to GDM. Any additional adverse events (including withdrawals due to adverse events) will be reported by the trial arm but will not be analysed through hypothesis testing.

### Additional analyses

As the women in the intervention are also asked questions related to whether they have seen the videos or attended the peer sessions if diagnosed with GDM, we will also perform a per-protocol analysis where only women who report seeing at least one of the educational films are analysed as part of the intervention arm. However, as one component of the intervention involves the professional development of doctors and nurses, women still may be impacted by the intervention through the doctors and nurses even if the women have not seen the video themselves. We will present the number and proportion of women in the intervention clinics who report having seen at least one video. Among women with GDM, we will also report the distribution of the number of times attending a group session (coded as a categorical variable, with levels 0, 1 or 2 times, 3 or 4 times, and 5 or more times).

As an additional data quality assessment to aid in our interpretation of results for adverse perinatal outcomes from the 32-week follow-up, we will compute the distribution in a number of weeks between the date of the last menstrual period reported at baseline and the date of delivery reported in the 32-week follow-up survey among women who reported that they were no longer pregnant (reported as minimum, 25th percentile, median, 75th percentile and maximum). Where available, we will also compare the expected delivery date reported at baseline to the date reported in the final follow-up survey. Finally, we will describe the distribution of computed gestational age of women at the time of completion of each follow-up survey. These checks will inform the strength of our assumption that all births reported at 32-week follow-up are preterm births, which in turn will affect how we interpret our adverse perinatal outcome analyses.

To account for the changes to the definition of co-primary outcome 1, we will also perform a sensitivity analysis wherein we restrict the sample to include only women who completed the 32-week follow-up after the 32-week follow-up questionnaire was revised to include additional questions about the timing of GDM screening (see the “Protocol updates” section). Note that this restriction will decrease the sample size and possibly lead to an underpowered analysis. Still, we expect it may be meaningful to describe how the prevalence of timely GDM screening differs in the two trial arms when excluding women who had to report this information several weeks after the end of their pregnancy.

We also will generate the following list of supplemental tables for each country:

- Supplemental Table 1: number of participants per health centre (including column for each stage of follow-up)
- Supplemental Table 2: demographic comparison of women lost to follow-up vs baseline covariates of women who completed all follow-up
- Supplemental Table 3: primary and secondary analysis results calculated using cluster-level summaries
- Supplemental Table 4: prevalence of individual components of composite adverse perinatal outcome

## Trial status and discussion

In Uganda, recruitment of trial participants began in May 2021 and ended in April 2022. In India, recruitment began in July 2021, and recruitment to the main trial ended in January 2022. Additional recruitment of women with GDM took place in India between April 2022 and December 2022 (see the “Protocol updates” section for details). Follow-up of the final participants is expected to occur in March 2023 in Uganda and May 2023 in India. This SAP was submitted for publication before the completion of follow-up in both countries. Analysis code will be developed and implemented following the submission of this SAP.

### Protocol updates

Since the publication of the original protocol in 2021 [7], there have been several important changes to the study protocol. These changes were predominantly made in order to mitigate the impact of COVID-19-related disruption on the study. All protocol amendments were initially approved by the LSHTM Ethics Committee and subsequently also approved by local ethics committees.

In the original protocol [7], the recruitment method was intended to be the same in India and Uganda: pregnant women were to be recruited from waiting areas at participating antenatal clinics. While this recruitment method was feasible throughout the study period in Uganda, in India, local guidance issued early in the COVID-19 pandemic prohibited women from waiting inside antenatal care facilities. BBMP, under whose jurisdiction we conducted the trial in Bangalore, India, mandated us to switch to remote recruitment using their maternal and child health (MCH database). Additionally, we had intended for the general awareness-raising film to be shown to pregnant women in waiting areas of intervention facilities. Given the high smartphone ownership in our Indian study setting, we proposed that these films could instead be shared with women remotely via web links sent via WhatsApp or similar. In Uganda, the films were able to be shared in antenatal waiting rooms as originally intended. These changes to the study protocol were approved in January 2022 (LSHTM REC amendment 4).

Following a review of in-trial data in March 2022 (blinding preserved), we noted that a considerably lower proportion (~ 2%) of women were reporting a GDM diagnosis in both sites. Our original sample size was based on a 5% difference in GDM diagnosis (5% vs 10% prevalence) which would have been impossible to detect at this prevalence. Following discussion with stakeholders and our Trial Steering Committee, we changed our co-primary outcome 1 to the proportion of women who report being *tested* for GDM between 24 and 32 weeks. Our original co-primary outcome 1 (the proportion of women reporting a GDM diagnosis at 32 weeks) was retained as secondary outcome 2. Additionally, our original secondary outcome (a composite endpoint consisting of a number of possible adverse perinatal outcomes) included caesarean delivery, but this was changed to include emergency caesarean delivery as the relevant component. This change was made in response to an unexpectedly high prevalence of elective (planned) caesarean sections in India, likely due to financial incentives. A further revision to the protocol was made to allow the self-management intervention for women with GDM to be delivered remotely in India, in line with previous amendments the introductory film for pregnant women to be viewed remotely. This change was a further attempt to mitigate the adverse impact of COVID-19-related restrictions on the trial in India. The changes to the study protocol described above were approved in April 2022 (LSHTM REC amendment 5).

Finally, as the prevalence of self-reported GDM was lower than we anticipated, this meant that we had difficulty recruiting enough women for the evaluation of the self-management intervention. In India, we undertook an additional recruitment drive, with the study team reaching out to local laboratories and affiliated services in order to recruit additional women with GDM, therefore increasing the likelihood that we would reach the sample size necessary to evaluate the self-management intervention. Unfortunately, a similar approach was not feasible in Uganda due to a lower prevalence of GDM screening. Women recruited through this targeted approach will not be included in the analysis of co-primary outcome 1 (GDM testing) and our secondary outcomes (composite perinatal outcome and GDM diagnosis). This change was also approved in April 2022 (LSHTM REC amendment 5).

Following the revision to the co-primary outcome from the proportion of women with self-reported GDM diagnosis to the proportion of women reporting GDM screening between 24 and 32 weeks of gestational age, an additional question was added to the 32-week follow-up questionnaire. In this original questionnaire, women were only asked about the timing of their *first* GDM test. The additional question asked women who reported receiving more than one test for GDM “Were any of these tests done between 24 and 32 weeks (6 and 8 months) of pregnancy?”. Note that this question was asked retrospectively of women who completed the 32-week follow-up before this question was added. Women will be considered as “screened for GDM at or after 24 weeks” if they reported any GDM screening that occurred between 24 and 32 weeks gestational age (or between 6 and 8 months gestational age, if reporting in months). If a woman reports her gestational age at screening in both weeks and months, the value in weeks will be used to determine the outcome due to the increased precision of this measurement.

Due to challenges related to staff funding, some women completed the postnatal questionnaire much later than intended. Furthermore, women who missed the window for the 32-week questionnaire were asked a modified version of these questions at the postnatal follow-up. A sensitivity analysis related to women’s gestational age (or weeks since the end of pregnancy) at each questionnaire is described in a previous section.

In the original protocol [7], co-primary outcome 1 (glycaemic control in women with GDM) was due to be assessed by both FBS and HbA1c. HbA1c was dropped from this outcome prior to the start of study recruitment. This decision was taken due as HbA1c is a measure of glycaemic control over the previous 2 to 3 months [18] and therefore would not capture any short-term impact of the intervention on glycaemic control. HbA1c was instead retained as an additional outcome.

## Data Availability

Not applicable.

## Abbreviations

BBMP: Bangalore Municipal Corporation
CONSORT: Consolidated Standards of Reporting Trials
GDM: Gestational diabetes mellitus
LMIC: Low- and middle-income country
MAR: Missing at random
OGCT: Oral glucose challenge test
PI: Principal investigator
PIS: Patient information sheet
SAP: Statistical analysis plan

## References

[1] Bellamy L, Casas J-P, Hingorani AD, Williams D (2009). Type 2 diabetes mellitus after gestational diabetes: a systematic review and meta-analysis. Lancet.

[2] Clausen TD, Mathiesen ER, Hansen T, Pedersen O, Jensen DM, Lauenborg J, Damm P. High prevalence of type 2 diabetes and pre-diabetes in adult offspring of women with gestational diabetes mellitus or type 1 diabetes: the role of intrauterine hyperglycemia. Diabetes Care. 2008;31(2):340–6. 10.2337/dc07-1596 18000174

[3] Buchanan TA, Xiang AH, Page KA (2012). Gestational diabetes mellitus: risks and management during and after pregnancy. Nat Rev Endocrinol.

[4] Alwan N, Tuffnell DJ, West J. Treatments for gestational diabetes. The Cochrane Library. 2009. 10.1002/14651858.CD003395.pub2 PMC7154381 19588341

[5] Atun R, Davies JI, Gale EAM, Bärnighausen T, Beran D, Kengne AP, Levitt NS, Mangugu FW, Nyirenda MJ, Ogle GD (2017). Diabetes in sub-Saharan Africa: from clinical care to health policy. Lancet Diabetes Endocrinol.

[6] Brown J, Alwan NA, West J, Brown S, McKinlay CJ, Farrar D, Crowther CA. Lifestyle interventions for the treatment of women with gestational diabetes. Cochrane Database of Syst Rev. 2017;(5). 10.1002/14651858.CD011970.pub2 PMC6481373 28472859

[7] Oakley LL, RD, Namara A, Sahu B, Nadal IP, Ana Y, Coombe H, Oteng-Ntim E, Seeley J, Nyirenda M, et al. Educational films for improving screening and self-management of gestational diabetes in India and Uganda (GUIDES): study protocol for a cluster-randomised controlled trial. Trials. 2021;22(1):501. 10.1186/s13063-021-05435-x PMC8320205 34321046

[8] Campbell MK, Piaggio G, Elbourne DR, Altman DG (2012). Consort 2010 statement: extension to cluster randomised trials. BMJ..

[9] Babu GR, Tejaswi B, Kalavathi M, Vatsala GM, Murthy GVS, Kinra S, Neelon SEB (2015). Assessment of screening practices for gestational hyperglycaemia in public health facilities: a descriptive study in Bangalore, India. J Public Health Res.

[10] Crowther CA, Hiller JE, Moss JR, McPhee AJ, Jeffries WS, Robinson JS (2005). Effect of treatment of gestational diabetes mellitus on pregnancy outcomes. N Engl J Med.

[11] Landon MB, Spong CY, Thom E, Carpenter MW, Ramin SM, Casey B, Wapner RJ, Varner MW, Rouse DJ, Thorp JM (2009). A multicenter, randomized trial of treatment for mild gestational diabetes. N Engl J Med.

[12] Vesco KK, Dietz PM, Bulkley J, Bruce FC, Callaghan WM, England L, Kimes T, Bachman DJ, Hartinger KJ, Hornbrook MC (2012). A system-based intervention to improve postpartum diabetes screening among women with gestational diabetes. Am J Obstet Gynecol.

[13] Clark HD, Graham ID, Karovitch A, Keely EJ (2009). Do postal reminders increase postpartum screening of diabetes mellitus in women with gestational diabetes mellitus? A randomized controlled trial. Am J Obstet Gynecol.

[14] Tierney M, O’Dea A, Danyliv A, Glynn LG, McGuire BE, Carmody LA, Newell J, Dunne FP (2015). Feasibility, acceptability and uptake rates of gestational diabetes mellitus screening in primary care vs secondary care: findings from a randomised controlled mixed methods trial. Diabetologia.

[15] Gamble C, Krishan A, Stocken D, Lewis S, Juszczak E, Doré C, Williamson PR, Altman DG, Montgomery A, Lim P (2017). Guidelines for the content of statistical analysis plans in clinical trials. JAMA.

[16] Schomaker M, Heumann C (2018). Bootstrap inference when using multiple imputation. Stat Med.

[17] Jakobsen JC, Gluud C, Wetterslev J, Winkel P (2017). When and how should multiple imputation be used for handling missing data in randomised clinical trials – a practical guide with flowcharts. BMC Med Res Methodol.

[18] Sherwani SI, Khan HA, Ekhzaimy A, Masood A, Sakharkar MK. Significance of HbA1c test in diagnosis and prognosis of diabetic patients. Biomarker Insights. 2016;11:BMI. S38440. 10.4137/BMI.S38440 PMC4933534 27398023

