# Correction: Statistical analysis plan and protocol updates for Gestational diabetes in Uganda and India: Design and Evaluation of Educational Films for Improving Screening and Self-management (GUIDES) trial

**DOI:** 10.1186/s13063-024-07959-4

**Published:** 2024-03-13

**Authors:** Nick Birk, Laura L. Oakley, Poppy A. C. Mallinson, Deepa R, Giridhara R. Babu, Mofat Nyirenda, Sanjay Kinra

**Affiliations:** 1 Department of Non-Communicable Disease Epidemiology, London School of Hygiene and Tropical Medicine, Keppel Street, London, WC1E 7HT UK; 2 Centre for Fertility and Health, Norwegian Institute of Public Health, Skøyen, P.O. box 222, N-0213 Oslo, Norway; 3 Global Health, Department of Population Medicine, College of Medicine, QU Health, Qatar University, Doha, Qatar; 4 MRC/UVRI and LSHTM Uganda Research Unit, Entebbe, Uganda

**Correction: Trials 24, 520 (2023)**

**https://doi.org/10.1186/s13063-023-07508-5**

Following publication of the original article [1], we have been notified that the 5th author’s affiliation was incorrect.

Corrected affiliation: Giridhara R Babu, Professor of Global Health, Department of Population Medicine, College of Medicine, QU Health, Qatar University.
